# Case Report: A Multimodal Treatment Approach Combining Multi-Target rTMS with a Modified, Largely Self-Administered, Clinician-Supervised Exposure and Response Prevention Protocol Using a Verbal Termination Cue in Two Patients with Severe Obsessive–Compulsive Disorder

**DOI:** 10.3390/jcm15187155

**Published:** 2026-09-15

**Authors:** Mohammad Mari, Markus Stingl, Bernd Hanewald, Christoph Mulert

**Affiliations:** Center for Psychiatry and Psychotherapy, Justus Liebig University Giessen, Klinikstrasse 36, 35392 Giessen, Germany; markus.stingl@psychiat.med.uni-giessen.de (M.S.); bernd.hanewald@psychiat.med.uni-giessen.de (B.H.); christoph.mulert@psychiat.med.uni-giessen.de (C.M.)

**Keywords:** obsessive–compulsive disorder, exposure and response prevention, repetitive transcranial magnetic stimulation, intermittent theta-burst stimulation, case report

## Abstract

**Background/Objectives**: Obsessive–compulsive disorder (OCD) is a chronic and disabling condition. Despite first-line treatment with cognitive behavioral therapy (CBT) including exposure and response prevention (ERP) and selective serotonin reuptake inhibitors (SSRIs), many patients remain severely symptomatic. This highlights the need for further research into innovative multimodal treatment strategies for OCD. We report two inpatients with severe OCD, comorbid depressive symptoms, and insufficient response to previous pharmacological and psychotherapeutic treatments. **Methods**: Both patients completed 30 multimodal treatment units over six weeks consisting of sequential multi-target repetitive transcranial magnetic stimulation (rTMS) targeting the left dorsolateral prefrontal cortex (DLPFC), right DLPFC, and supplementary motor area (SMA), combined with a modified, largely self-administered, clinician-supervised exposure and response prevention protocol. **Results**: Both patients demonstrated large observed reductions in OCD symptom severity. Y-BOCS total scores decreased from 29 to 11 in Patient 1 and from 29 to 10 in Patient 2, corresponding to reductions of 62.1% and 65.5%, respectively, with improvements apparent after approximately four weeks and largely maintained at follow-up. Depressive symptoms also improved, and no clinically relevant adverse effects were observed. **Conclusions**: These cases provide an initial description of the feasibility and tolerability of this specific protocol architecture but do not establish efficacy, long-term durability, or the contribution of individual components. The protocol-level novelty lies in integrating a repeated three-target TBS schedule with a response-limited, cue-terminated exposure procedure, a combination not previously described in the OCD neuromodulation literature.

## 1. Introduction

Obsessive–compulsive disorder (OCD) is a common, chronic, and highly disabling psychiatric disorder characterized by intrusive, unwanted thoughts, urges, or mental images (obsessions) and repetitive behaviors or mental acts (compulsions) [1,2]. These symptoms are typically associated with significant functional impairment, reduced quality of life, and frequent comorbidity with depressive and anxiety disorders [3,4].

According to current evidence-based recommendations, cognitive behavioral therapy (CBT) with ERP is considered the preferred first-line psychotherapeutic treatment for OCD; pharmacologically, selective serotonin reuptake inhibitors (SSRIs) are recommended as the first-line treatment, while clomipramine is typically considered a second-line option due to comparable efficacy but a more unfavorable side effect profile [5,6,7]. Nevertheless, the management of treatment-resistant OCD persists as a significant challenge in clinical psychiatry. Clinical studies indicate that approximately 40–60% of patients treated with SSRIs exhibit a clinically relevant reduction in obsessive–compulsive symptoms, while the rest of the patients respond only partially or not at all [6,8]. Some reviews suggest that response rates to SSRI monotherapy do not exceed approximately 50% overall [7].

Even when first-line pharmacotherapy is combined with cognitive behavioral therapy, a substantial proportion of adults with OCD fail to achieve sufficient symptom remission or do not respond adequately to these standard interventions, with overall rates of resistance reported to reach up to 60% [9,10,11,12]. These persistent treatment limitations underscore the clinical necessity of exploring novel, multimodal therapeutic strategies.

rTMS has become an established, safe, and tolerable neuromodulatory intervention in major depressive disorder and has more recently emerged as a promising therapeutic option for OCD [13,14,15], with recent studies suggesting that multi-target rTMS protocols may lead to clinically relevant symptom reductions [16,17].

An increasing number of neuroimaging studies suggest that dysfunctional cortico-striato-thalamo-cortical (CSTC) circuits play a role in the pathophysiology of OCD [18,19,20]. Among the explored cortical stimulation sites, the bilateral dorsolateral prefrontal cortex (DLPFC) and the (pre-)supplementary motor area (SMA) have been identified as particularly relevant cortical nodes in OCD, as they are involved in executive control, response inhibition, and repetitive action patterns, thereby representing promising therapeutic targets for neuromodulatory interventions such as rTMS [21,22,23,24]. Building on this neurobiological rationale, and informed by a prior case series demonstrating the feasibility and safety of targeting multiple cortical regions implicated in OCD pathophysiology within a single treatment session [16], we selected bilateral DLPFC and SMA as stimulation targets for the multi-target rTMS protocol described in this report. While the general targeting strategy was informed by this prior report, the specific stimulation parameters, sequencing, and session structure applied in our protocol were adapted and further specified for this case, as detailed in Section 2.

In parallel, there is considerable evidence for the effectiveness of intensive, concentrated formats of ERP in OCD. In a randomized controlled trial, Launes et al. (2019) demonstrated that a concentrated ERP format delivered over four consecutive days was superior to both self-help and waiting-list conditions [25]. These results have been replicated in recent studies emphasizing the relevance of concentrated ERP in OCD [26]. Nevertheless, a notable sub-group of patients does not achieve clinically significant improvement after ERP or discontinues treatment before completion [27]. In a recent meta-analysis, Yan et al. (2022) therefore explicitly supported the rationale of combining ERP with other therapeutic methods [28]. In this sense, recent clinical studies have provided increasing evidence for the effectiveness of multimodal therapeutic approaches combining rTMS with cognitive behavioral therapy in OCD patients [29].

Based on the converging evidence supporting both cortical neuromodulation targeting key OCD-implicated brain regions and intensive exposure-based treatment of OCD, we implemented a multimodal treatment strategy integrating rTMS with exposure-based learning. However, previous case reports have described sequential multi-target rTMS involving the bilateral DLPFC and SMA [30], and other studies have combined rTMS with cognitive-behavioral treatment within structured multimodal protocols [29]. The present report therefore does not claim novelty for multi-target stimulation or rTMS–psychotherapy co-treatment per se. Rather, its protocol-level novelty lies in integrating (i) a repeated two-sessions-per-day theta-burst stimulation schedule comprising sequential left DLPFC iTBS, right DLPFC cTBS, and SMA cTBS, followed 60 min later by a second left DLPFC iTBS session, with (ii) a clinician-supervised, largely self-administered exposure procedure incorporating expectation testing, a single pre-specified completion check, a fixed verbal termination cue (“END”), and subsequent abstention from further checking. To our knowledge, this exact protocol architecture has not previously been described in severe OCD.

Here, we present two cases of severe OCD treated with this combined approach: Patient 1 demonstrated treatment non-response corresponding to at least Level II of the operational staging model for levels of non-response proposed by Pallanti et al., with persistent clinically significant symptoms despite an adequate SSRI trial and documented exposure and response prevention (ERP) without meaningful clinical improvement [31], whereas Patient 2 presented with severe OCD and insufficient symptom control despite multiple previous interventions.

The aim of this report is to describe the clinical rationale and provide an operational description of the integrated protocol, and to document its feasibility, tolerability, and observable clinical course in two cases. It is not intended to establish efficacy or to determine the contribution of individual treatment components; robust conclusions regarding efficacy would require prospective controlled trials.

Case reports

Both patients were treated at the Center for Psychiatry and Psychotherapy at the University Hospital of Giessen and Marburg (UKGM), Justus Liebig University Giessen, Germany, and received standard multimodal inpatient psychiatric care, including medical and psychiatric assessment, medication management, nursing support, individual psychotherapy, group psychotherapy, art therapy and movement therapy as part of their treatment. These components focused on general stabilization, symptom management, and supportive treatment rather than on OCD-specific mechanisms. In particular, no systematic exposure and response prevention or other OCD-specific behavioural interventions were provided outside the treatment protocol.

Case 1

A 27-year-old woman presented to our university hospital as an emergency case with a severe exacerbation of obsessive–compulsive disorder, present since 2022, with insufficient response to previous treatment attempts. The OCD symptoms were dominated by pronounced compulsive checking (doors, kitchen stove, water faucets) that took up to several hours a day, as well as compulsive washing, which led to significant limitations in everyday functioning, including withdrawal from social activities and hobbies. In the weeks preceding inpatient admission, the patient’s clinical condition progressively deteriorated, accompanied by a severe depressive episode and recurrent suicidal ideation. Given this presentation, the patient was initially admitted to an acute psychiatric ward for close monitoring and crisis stabilization. Following stabilization, a structured psychiatric evaluation confirmed the absence of active suicidal intent, a reliable capacity to distance herself from suicidal thoughts, and reliable help-seeking behavior. Only after this confirmation of clinical stability was the intensive modified response-prevention protocol combined with multi-target rTMS initiated. Suicidal thoughts and mood were reassessed throughout treatment as part of routine ward safety monitoring, with predefined criteria for treatment interruption in the event of clinical deterioration.

Before the current inpatient admission, the patient had undergone multiple inpatient treatment episodes over several years, including psychotherapy and pharmacological interventions, without achieving any clinically significant improvement in OCD symptoms. Paroxetine was titrated to a maximum dose of 60 mg/day and administered for a total of 13 weeks; it was subsequently discontinued because of insufficient clinical benefit and at the patient’s request.

The most recent pharmacological intervention prior to rTMS consisted of a combination of clomipramine 225 mg/day (initiated in 03/2025) and risperidone 2 mg/day (initiated in 06/2025). Both medications were largely maintained during the multi-target rTMS treatment course; however, despite adequate treatment duration and combined pharmacotherapy, this treatment did not result in clinically meaningful symptom reduction. The available medical records documented previous ERP sessions without clinically meaningful benefit. However, the total treatment dose, degree of therapist supervision, adherence, and fidelity to complete response prevention could not be reconstructed retrospectively. Taken together, the available treatment history was consistent with treatment non-response corresponding to at least Level 2 of the operational staging model outlined above (for a compact overview of the clinical timeline for Patient 1, see Table 1).

Due to persistent severe symptoms (Y-BOCS baseline: 29 points) and comorbid depression (BDI-II baseline: 50 points), and following careful clinical evaluation and informed consent, multimodal treatment consisting of multi-target rTMS and intensive modified response-prevention protocol was initiated.

Case 2

A 25-year-old man was admitted to our university hospital due to severe OCD with a comorbid major depressive episode. Since 2020, the patient had experienced recurrent depressive episodes and obsessional thoughts, and during the 6 months before admission, these symptoms progressed to pronounced compulsive checking rituals, which occupied several hours daily and led to significant functional impairment. He repeatedly checked electrical outlets, doors, light switches, and his car, sometimes checking whether his car was locked up to 20 times. He also sought frequent reassurance from his parents. Persistent uncertainty and fear of insufficient checking resulted in repeated returns to his apartment and considerable sleep-onset difficulties.

The patient had undergone several previous inpatient treatments and multiple pharmacological interventions, including escitalopram, sertraline, amisulpride, quetiapine, mirtazapine, trazodone, and risperidone. These treatments were either discontinued due to adverse effects (e.g., hyperprolactinemia, intolerable side effects) or found to be insufficiently effective, resulting in successive modifications of psychopharmacological therapies across different hospitalizations.

The most recent pharmacological intervention prior to rTMS consisted of lithium 1350 mg/day (initiated in 05/2025), amitriptyline 200 mg/day (initiated in 05/2025), and aripiprazole 15 mg/day (added in 01/2026 as augmentation). Amitriptyline was increased to 200 mg/day during the treatment period, whereas the other medication doses remained unchanged. Despite sufficient treatment duration and combination therapy, this regimen had not led to any clinically relevant improvement (for a compact overview of the clinical timeline for Patient 2, see Table 2). Considering the persistent severe symptoms (Y-BOCS baseline: 29 points) and the comorbid severe depressive episode (BDI-II baseline: 37 points), and following careful evaluation and informed consent, multimodal therapy consisting of multi-target rTMS and intensive modified response-prevention protocol was initiated.

## 2. Materials and Methods

### 2.1. Diagnostic Assessment

Both patients underwent a comprehensive clinical psychiatric assessment, including psychiatric history, mental-status examination, review of available medical records, and assessment of obsessive–compulsive symptom dimensions and severity using the Yale–Brown Obsessive–Compulsive Scale (Y-BOCS) and its Symptom Checklist. Diagnoses of obsessive–compulsive disorder and comorbid depressive disorder were established by a board-certified psychiatrist according to ICD-10 diagnostic criteria. Apart from OCD and comorbid depressive disorder, no additional psychiatric diagnoses were documented at the time of rTMS initiation. Y-BOCS assessments were conducted as part of routine clinical care by trained clinicians involved in the patients’ inpatient treatment. The assessors were therefore neither independent nor blinded to treatment or assessment time point. No structured diagnostic interview was administered.

### 2.2. Therapeutic Intervention

The intervention was conceived as a coordinated daily protocol rather than as the parallel delivery of two independent treatments. Its distinguishing architecture comprised repeated sequential stimulation of three cortical targets, a second daily left-DLPFC session, and a standardized four-step exposure exercise completed largely without therapist presence but reviewed daily by a clinician.

### 2.3. rTMS Parameters

Stimulation was applied using a MagPro X100 stimulator and a figure-of-eight coil (MCF-B70), both manufactured by MagVenture GmbH (Willich, Germany). The treatment protocol comprised 30 treatment units, completed over a period of 6 weeks. Each unit consisted of two rTMS sessions per day, with a 60-min interval between sessions. The stimulation intensity ranged from 90% to 100% of the individually determined resting motor threshold (RMT). The resting motor threshold was determined by delivering single magnetic pulses over the left primary motor cortex and identifying the minimum stimulator intensity required to elicit a visible movement of the right hand (e.g., a brief twitch); this confirmed that the magnetic stimulus effectively reached the underlying cortical neurons, consistent with the previously described procedure [32]. RMT was reassessed periodically throughout the treatment course and additionally whenever clinically indicated, particularly following changes in clinical status, sleep patterns, or concomitant medication considered likely to influence cortical excitability or the seizure threshold, in accordance with current consensus recommendations [33]. However, the exact dates and frequency of reassessment could not be reconstructed reliably from the retrospective clinical documentation.

Stimulation intensity was individually titrated between 90% and 100% RMT, depending on tolerability. In patient 1, the intensity was increased from 90% to 100% RMT across all three target areas over the course of treatment, whereas in patient 2, it was maintained at 90% RMT throughout the entire treatment period due to tolerability considerations. Both intensities remained within the range considered standard in current clinical practice, as a systematic review of 3784 articles reported that the majority of published rTMS protocols use stimulation intensities ranging from 80% to 120% of the motor threshold [34].

Before rTMS treatment, both patients underwent a structured safety screening based on the international TMS safety guidelines [35,36], including a systematic assessment of seizure history, family history of epilepsy, prior head trauma, intracranial metal implants, and concomitant medications known to lower seizure threshold. In both patients, structural brain MRI and EEG had additionally been performed as part of routine inpatient diagnostic work-up, and showed no findings that would represent a contraindication for rTMS. Possible rTMS-related adverse effects were systematically assessed in each daily session through direct clinical observation by the treating team and clinician-elicited patient reports. No clinically significant adverse events, including no seizures, were observed in either of the two patients throughout the entire course of treatment.

**Figure 1:**
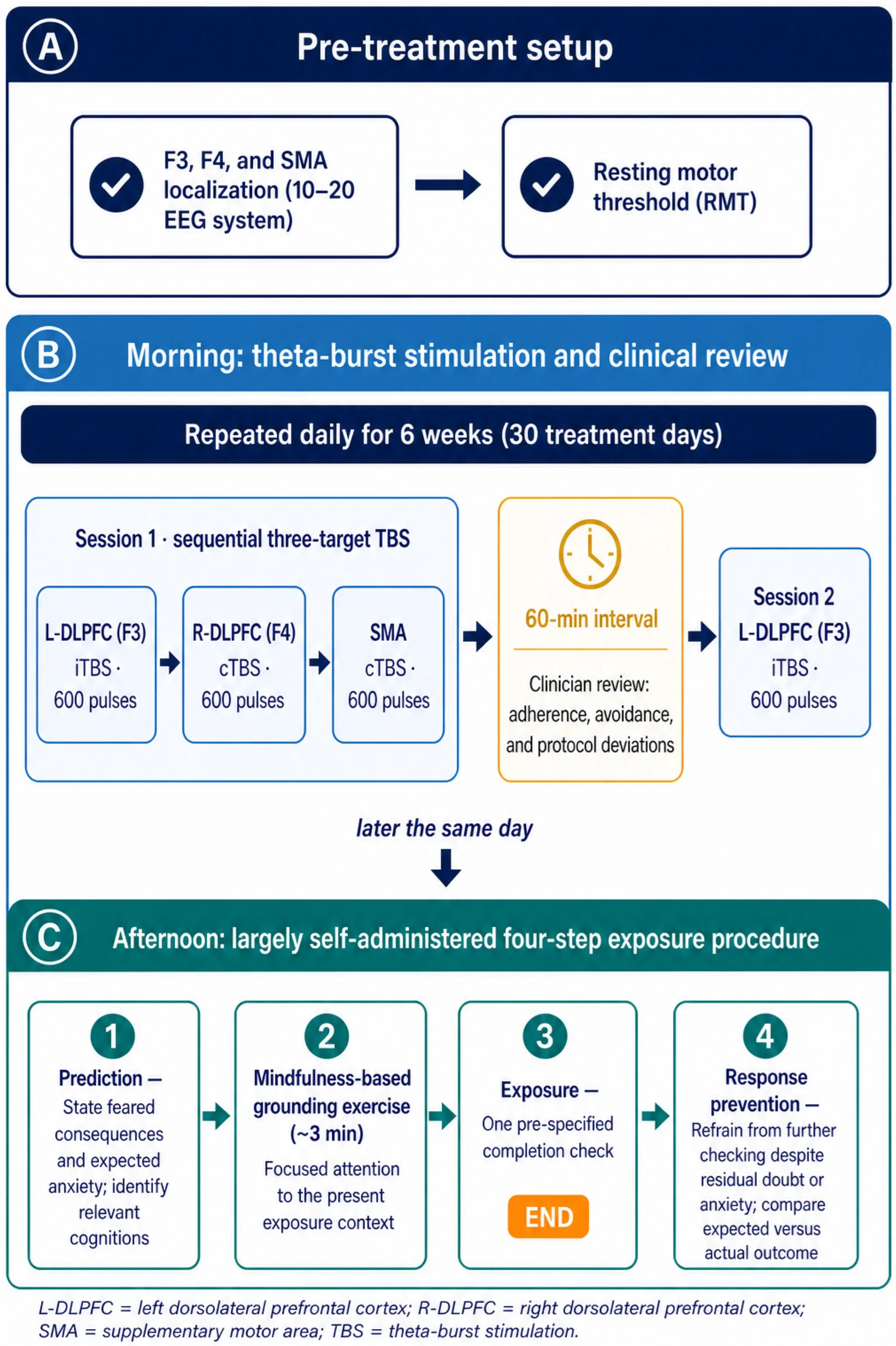
Overview of the multi-target rTMS treatment protocol. Each treatment day consisted of two rTMS treatments with a 60-min interval. Session 1 targeted three cortical regions sequentially: the left dorsolateral prefrontal cortex (L-DLPFC), the right dorsolateral prefrontal cortex (R-DLPFC), and the supplementary motor area (SMA). Session 2 consisted of iTBS applied exclusively over the L-DLPFC. All three stimulation targets were localized using established 10–20 EEG system coordinates prior to the first treatment session. The resting motor threshold (RMT) was determined prior to the start of treatment. Later the same day, patients completed a largely self-administered, modified exposure-based procedure comprising four steps: symptom prediction, a brief mindfulness-based grounding exercise, a pre-specified completion check, and response prevention.

In the first daily session, three brain regions were sequentially stimulated:

(1) The left dorsolateral prefrontal cortex (L-DLPFC, position F3) was stimulated using intermittent theta-burst stimulation (iTBS; 600 pulses, in a burst-firing pattern consisting of 3 pulses at 50 Hz, in 2-s trains with 8-s intertrain intervals), with a total duration of 3 min 9 s [37].
(2) The right dorsolateral prefrontal cortex (R-DLPFC, position F4) was stimulated with continuous theta-burst stimulation (cTBS; 600 pulses; total duration 40 s). To stimulate the left and right DLPFCs, the figure-8 coil was centered over the manually identified positions F3 and F4, respectively, and oriented at approximately 45° relative to the midsagittal plane, a clinically used procedure for DLPFC stimulation [38,39].
(3) The supplementary motor area (SMA) was stimulated using cTBS (600 pulses; total duration 40 s). The second daily session consisted of iTBS (600 pulses) over the left DLPFC only. Accordingly, 2400 pulses were administered per treatment day, resulting in a cumulative dose of 72,000 pulses over the 30 treatment days. An overview of the rTMS treatment protocol is presented in Figure 1.

Within the first daily session, the L-DLPFC, the R-DLPFC, and the SMA were stimulated sequentially. Brief intervals of up to approximately 5 min between the individual target areas were required for coil repositioning and alignment over the previously determined scalp locations. These intervals reflected procedural preparation and were not fixed protocol-defined intervals.

Positions F3 and F4 were identified manually according to the international 10–20 EEG system. The SMA was determined as the point located 15% of the inion–nasion distance anterior to the vertex (Cz) on the sagittal line; the coil was positioned along the sagittal midline with the handle oriented toward the occipital region [40].

### 2.4. Intensive Modified Response-Prevention Protocol

The behavioral component differed from conventional ERP in three predefined respects: exposure was completed largely without therapist presence after structured preparation; one pre-specified completion check was permitted; and a fixed verbal cue (“END”) marked the transition to complete abstention from any further checking despite residual anxiety, doubt, or tension. Daily clinician review focused on adherence, avoidance, and protocol deviations.

The behavioral intervention consisted of a structured 4-step, modified response-prevention protocol, practiced largely independently after preparatory instruction and with regular daily review during the rTMS treatment phase. Before the start of the protocol, each patient participated in an individual preparatory session lasting approximately 90 min, which included a symptom analysis, a severity estimation, and comprehensive psychoeducation explaining the rationale for the combination of multi-target rTMS with the intensive, modified response-prevention protocol, as well as the key dysfunctional cognitive patterns associated with OCD, including threat overestimation, catastrophizing, intolerance of uncertainty, and inflated responsibility. Following this preparatory session, exposure exercises were performed once daily by each patient in the afternoon, while the two daily rTMS sessions were conducted in the morning, allowing each exercise to be completed without time pressures imposed by the rTMS treatment schedule. Each exposure exercise focused on clinically relevant compulsive rituals identified in collaboration with the treating physician, with the specific target and duration adapted individually to the patient’s symptom presentation and tolerance. Adherence and subjective experience were assessed daily during a structured debriefing conducted by the treating clinician in the interval between the two rTMS sessions; this assessment included verification that the exercise had been performed and identification of any avoidance behaviors or rule modification. No additional homework assignments were prescribed beyond the once-daily self-administered exposure exercise. No formal, standardized adherence-monitoring instrument was used; adherence was assessed clinically based on the patient’s verbal account during these daily reviews.

The daily protocol consisted of four structured steps:

- First, before each exposure, patients were instructed to explicitly formulate their own expectations regarding the current situation (i.e., feared consequences and expected anxiety intensity) and to identify relevant dysfunctional cognitions. After exposure, patients compared their initial expectations with their actual experience.
- Second, immediately before exposure, patients completed a brief mindfulness-based reality check of approximately 3 min in the exposure context, intended to promote objective awareness of the situation and to support corrective appraisals such as “Fear is a feeling, not reality.” It further served to interrupt the rapid transition from intrusive thoughts and anxiety to habitual neutralizing responses before exposure began. The exercise was therefore designed more as a brief attentional anchoring strategy preceding exposure, rather than as reassurance or a safety behavior.
- Third, instead of allowing unlimited repetition, patients were instructed to perform the control or repetitive behavior only once in a single, pre-defined trial, thereby creating a predefined, externally specified behavioral endpoint in place of the unreliable internal termination signal characteristic of OCD. Immediately after the completion of this single trial, the previously established verbal cue (“END”) was verbalized audibly by the patient, typically in a quiet tone, to terminate the response cycle. This verbal termination cue was not intended to satisfy or reinforce the compulsion, but rather to replace the missing subjective sense of completion with an objective behavioral endpoint, with the intention to prevent the endless repetition of uncontrolled compulsive sequences. The cue was used consistently throughout the entire treatment period to condition an association between the response initiation and its conscious, externally verified termination.
- Fourth, patients first confronted the anxiety-provoking stimulus; following the single, pre-specified completion check and application of the termination cue (“END”), patients left the situation without performing any further checking or repetitive behavior, regardless of any remaining anxiety, doubt, or tension, and then completed a brief post-exposure reflection.

During the preparation of this manuscript, the authors used Perplexity (Perplexity AI, Inc.; 115 Sansome Street, Suite 900, San Francisco, CA 94104, USA; https://www.perplexity.ai; accessed on 7 September 2026) for grammar checking and language polishing of selected passages of the manuscript text. Figure 1 was independently designed and created in its entirety by the authors; Perplexity was additionally used solely to enhance the resolution and visual clarity of the already completed figure. All scientific content, data interpretation, and figure design and content decisions were made entirely by the authors. The authors reviewed and verified all AI-assisted output and take full responsibility for the accuracy and integrity of the published content.

## 3. Results

Both patients completed 30 treatment units over a total period of 6 weeks and showed marked reductions in OCD severity, accompanied by patient-reported functional improvement in daily life.

Patient 1 showed a reduction in Y-BOCS score from 29 at baseline to 12 after 20 sessions and to 11 at treatment completion, corresponding to a 62.1% reduction from baseline. At the 4-week follow-up, the Y-BOCS score had further decreased to 9, suggesting maintenance of symptom improvement at the 4-week follow-up.

Patient 2 showed a reduction in Y-BOCS score from 29 at baseline to 11 after 20 sessions and to 10 at treatment completion, representing a 65.5% reduction from baseline. At the 4-week follow-up, the Y-BOCS score was 13, indicating partial maintenance of improvement relative to baseline, although symptoms increased slightly compared with treatment completion.

Depressive symptoms also improved, particularly in Patient 1, whose BDI-II score decreased from 50 at baseline to 15 after 20 treatment units and 8 after 30 treatment units. Patient 2 showed a decrease in BDI-II score from 37 at baseline to 20 after 30 treatment units. At the four-week follow-up, depressive symptomatology was reassessed using a structured clinical psychiatric interview covering the core symptom domains of depression, including depressed mood, anhedonia, sleep disturbance, energy levels, concentration difficulties, and suicidal ideation, rather than through a repeated standardized instrument. Based on this clinical assessment and in comparison with each patient’s discharge presentation, no re-emergence or worsening of these core depressive domains was observed in either patient. However, the BDI-II was not re-administered at this time point, and maintenance of the previously observed reduction in depressive symptoms could therefore not be evaluated quantitatively; this qualitative clinical observation cannot substitute for a standardized, quantifiable assessment and should be interpreted with corresponding caution. As the primary focus of the four-week follow-up was the durability of obsessive–compulsive symptom improvement, depressive symptomatology was assessed only as a secondary clinical parameter at this time point. No clinically relevant adverse effects were reported during treatment. An overview of all outcome measures at each assessment point is provided in Table 3.

## 4. Discussion

This two-case report illustrates the feasibility and tolerability of a multimodal protocol combining sequential multi-target rTMS with a structured, therapist-supervised, largely self-administered, modified response-prevention protocol in patients with severe OCD. Both patients showed marked reductions in Y-BOCS scores over six weeks, with improvements largely maintained at short-term follow-up. The clinical relevance of optimizing the treatment of OCD is further underscored by the World Health Organization’s recognition of OCD as one of the ten most disabling mental disorders and as being associated with a significant reduction in quality of life, work performance and social participation [41].

Over the past several decades, neurobiological studies have investigated the pathophysiology of OCD and have consistently demonstrated dysfunction in the cortico-striato-thalamic-cortical (CSTC) network in patients with OCD [42,43].

More recent functional connectivity analyses have reported particularly altered or reduced connectivity within sensorimotor CSTC loops in OCD compared to healthy controls [44,45]. Going beyond the classical CSTC model, recent meta-analyses and systematic reviews have provided evidence of dysregulation, mostly characterized by lower connectivity within and between the networks of the so-called triple-network model, including the default mode network (DMN), the fronto-parietal network (FPN), and the salience network (SN) in patients with OCD; such connectivity alterations may be associated with cognitive inflexibility and difficulties in shifting from intrusive, self-referential thoughts to target-oriented behavior [44,46].

In light of these findings, the left DLPFC is of special clinical relevance as a central node of the fronto-parietal network [47] and as a key node within the CSTC circuits involved in the pathophysiology of OCD [48]. Several neuroimaging studies have repeatedly shown dysregulated activation patterns in the left DLPFC as well as reduced functional connectivity within dorsal-cognitive CSTC loops in patients with OCD [49,50].

Since the left DLPFC is one of the most well-validated and most commonly used stimulation targets in rTMS therapy for depression [51] and, at the same time, has a key role in executive control and top-down inhibition of subcortical structures, this provides a dual neurobiological rationale for both OCD symptoms and the commonly comorbid depressive symptoms [52]. Given this background, we applied an excitatory iTBS protocol over the left DLPFC as part of our multimodal treatment approach, consistent with previously described effects of rTMS on prefrontal–striatal circuitry, with the aim of potentially contributing to the modulation of impaired top-down control and disrupted fronto-parietal control network activity.

In addition, an inhibitory continuous theta-burst stimulation (cTBS) was applied over the right DLPFC. The combination of iTBS (left DLPFC) and cTBS (right DLPFC) represents a well-studied bilateral protocol, which was investigated in patients with depressive disorders and found to be safe, feasible, and superior to sham in a randomized controlled pilot study [53] as well as in a multicenter randomized controlled trial [54]. Furthermore, several studies provide evidence for the therapeutic relevance of the right DLPFC as a stimulation target in OCD [55]. In a double-blind RCT, Elbeh et al. (2016) demonstrated that 1-Hz rTMS over the right DLPFC produced sustained reductions in obsessive–compulsive symptoms with additional improvements in anxiety symptoms [56]. Comparable effects were reported in the study by Khedr et al. (2022), in which low-frequency rTMS over the right DLPFC produced clinically relevant improvements in obsessive–compulsive symptoms compared to sham stimulation [24].

As the third stimulation target, the supplementary motor area (SMA) was included because the pre-SMA is centrally involved in response inhibition and voluntary behavioral change, functions that are fundamentally impaired in OCD [57]. Consistent with these functional impairments, imaging connectivity studies show that patients with OCD exhibit increased functional connectivity between the pre-SMA and the inferior frontal gyrus (IFG), which is associated with greater disruption of motor response inhibitory control [58]. These two regions are considered key nodes within cortico-striato-thalamic circuits, which are involved in motor response inhibition [59]. The therapeutic relevance of the SMA as a rTMS target in OCD has been demonstrated by several randomized controlled trials. Singh et al. (2025) reported a 35.6% reduction in Y-BOCS scores following eight weeks of low-frequency rTMS (1 Hz) over the right SMA [60]. Converging evidence from neuromodulation and neuroimaging studies further identifies the pre-SMA as a key node within inhibitory control networks in OCD and as a promising target for neuromodulatory interventions, particularly rTMS-based ones [61,62]. However, several case reports and case series suggest that protocols sequentially targeting multiple neural circuits implicated in OCD pathophysiology within the same treatment course may achieve additive or synergistic clinical benefit compared with single-target approaches [16,30,63]. As sequential three-target protocols of this kind are relatively novel, to the best of our knowledge, no adequately powered controlled trial has directly compared such three-target rTMS protocols with single-target approaches in OCD, and this rationale should therefore be regarded as hypothesis-generating rather than empirically established.

Nevertheless, each of the targets selected for this protocol is independently supported by controlled evidence: a network meta-analysis found that low-frequency rTMS (LF-rTMS) over the right DLPFC and SMA, as well as high-frequency rTMS (HF-rTMS) over the left DLPFC, showed greater efficacy compared with placebo treatment, with comparable tolerability across all protocols. However, the authors note that, according to the GRADE framework, the certainty of evidence for the overall ranking of efficacy and tolerability was rated as very low and low, respectively [23]. This limited certainty for individual targets provides additional rationale for combining established targets within a single protocol, an approach that is further supported by case reports in which the sequential stimulation of three target regions (bilateral SMA and the left and right DLPFCs) within a single session using combined LF-rTMS/iTBS protocols resulted in clinically significant symptom improvements in treatment-resistant OCD [30]. A 60-min inter-session interval was implemented between consecutive rTMS sessions, in accordance with accelerated stimulation protocols that have used similar intervals. Recent studies suggest that inter-session intervals of approximately 60–90 min are required to enable cumulative long-term potentiation-like effects [14,64]. The 60-min inter-session interval was chosen in line with accelerated stimulation protocols suggesting that intervals in this range may support cumulative plasticity-related effects. However, whether this interval contributed specifically to the clinical course observed here cannot be determined.

The rationale for the sequential combination of rTMS therapy and modified response-prevention protocol is based on complementary clinical and mechanistic considerations. From a clinical perspective, this multimodal strategy is supported by emerging studies combining rTMS with ERP or CBT-based interventions. Fitzsimmons et al. (2025) demonstrated that rTMS administered immediately before ERP in treatment-resistant OCD was associated with clinically significant symptom reduction [65]. From a mechanistic standpoint, this strategy is supported by the priming hypothesis, according to which rTMS modulates the relevant fronto-striatal circuits via long-term potentiation (LTP) and long-term depression (LTD)-like mechanisms of plasticity and metaplasticity, thereby enabling subsequent exposure-based learning inputs to be processed more efficiently [66,67]. Unlike prior rTMS-ERP protocols in which stimulation was delivered immediately before exposure to prime the relevant neural network, the present protocol used morning stimulation sessions followed by a later, largely self-administered exposure exercise. It should therefore be interpreted as a coordinated same-day treatment schedule rather than as a direct test of immediate rTMS priming of exposure learning. Given that priming effects of rTMS on cortical excitability are known to be time-window-dependent, whether this temporal separation preserves, attenuates, or is unrelated to any putative priming effect on exposure-based learning remains unknown and should be addressed in future controlled studies.

The structured modified response-prevention protocol was designed for patients who may not be able to perform exposure exercises under direct therapist supervision, whether due to logistical reasons, shame-related obsessional content, or limited therapeutic resources. Accordingly, patients received comprehensive instruction in the protocol, while adherence and implementation were reviewed daily during the interval between the two rTMS sessions, rather than during the exposure exercises themselves. This approach represents a pragmatic compromise between the clinic’s supervisory capacity and the aim of fostering patient autonomy in symptom management, rather than an attempt to replicate therapist-guided exposure as implemented in controlled ERP studies. The adherence was assessed based on patients’ self-reports alone, rather than through direct observation or standardized monitoring tools.

The integration of a brief mindfulness-based reality check immediately before exposure was intended to reduce anticipatory arousal and promote present-moment awareness, thereby interrupting the rapid transition from intrusive thoughts and anxiety to habitual neutralizing responses. This exercise was not intended to provide reassurance or to replace the comparison between expected and actual outcomes, which occurred during and after exposure. A theoretical possibility remains that any component preceding exposure could, in principle, acquire a reassuring function; however, no clinical indications of increased reliance, avoidance, or reassurance-seeking related to this exercise were observed in either patient. This rationale is grounded in the OCD-relevant cognitive model, in which overestimation of threat and its consequences is considered a central dysfunctional belief domain [68].

This rationale is consistent with evidence linking mindfulness-based interventions to altered amygdala–prefrontal connectivity and improved emotion regulation [69,70] and thus provides a plausible, though in the present cases untested, neurobiological rationale for this component.

Another specific feature of OCD psychopathology that was taken into account in the design of this modified response-prevention protocol is the frequently observed impairment in response inhibition; OCD patients consistently exhibit prolonged stop-signal reaction times (SSRTs), reflecting impaired inhibitory control over ritualistic responses [71,72]. This impairment in inhibitory control is closely linked to the phenomenon known as “not just right experiences”, a subjective feeling of incompleteness reported by the majority of OCD patients and considered a central phenomenological driver of repetitive correction and checking rituals [73,74].

The defining behavioral modification was not self-administration alone but the transition from one permitted, pre-specified completion check to complete response prevention, marked by a fixed verbal termination cue (‘END’). The cue was intended to provide an externally specified action endpoint while preserving exposure to unresolved doubt and residual anxiety. Because one completion check was permitted before this transition, the procedure as a whole did not constitute complete response prevention from the outset in the conventional ERP sense. This design was informed by models proposing that compulsive behavior in OCD may be maintained by a deficient internal completion signal, a diminished subjective sense that a security-related action has been sufficiently performed, rather than by threat avoidance alone [75]. This mechanism may be particularly relevant to preventive rituals, because the non-occurrence of the anticipated threat provides no observable external signal that the action has been completed. Consequently, patients may rely more heavily on an already impaired internal sense of completion [76]. The externally defined cue was accordingly conceptualized as a fixed, unambiguous external reference point that patients could mentally draw on during subsequent urges to re-check or repeat the action, functioning as a structural termination marker rather than as a mechanism to reduce perceived threat or provide reassurance. We acknowledge that whether the “END” cue facilitated disengagement from repetitive checking or instead functioned as a safety signal was not formally evaluated, and potential cue dependence was not prospectively assessed.

In both cases, the observed symptom reduction appeared early and reached a clinically meaningful level after approximately 4 weeks. Scores at 4 weeks were already close to final values at 6 weeks, suggesting possible early stabilization of the observed symptom reductions during treatment. This observation is clinically interesting because it may inform future individualized treatment durations, but it cannot be interpreted definitively in an uncontrolled case report. At treatment completion, both patients reported substantial subjective relief, consistent with the objectively measured improvements on standardized rating scales. At short-term follow-up assessment conducted four weeks after the end of treatment, both patients maintained their improvement, with Y-BOCS scores of 9 (Patient 1) and 13 (Patient 2), suggesting that symptom improvements were largely maintained over the short follow-up period; however, definitive conclusions regarding long-term durability require confirmation in controlled prospective studies.

These findings must be interpreted within the limitations of an uncontrolled case report of two patients. Because both patients received a multimodal inpatient treatment package, the observed symptom reductions cannot be attributed with certainty to rTMS, the exposure-based protocol, medication, hospitalization, or any other individual component in isolation. In addition, stimulation targets were manually localized using scalp-based anatomical landmarks according to the international 10–20 EEG system; MRI-guided neuronavigation was not used. Therefore, interindividual variability in the correspondence between locations on the scalp and the underlying cortical targets cannot be excluded. Furthermore, since this case report did not include neuroimaging, functional connectivity, electrophysiological, or other neurophysiological outcome measures, it cannot establish whether stimulation of the intended cortical target produced the proposed effects at the circuit level. The neurobiological framework presented here should therefore be interpreted as a theoretical, literature-based rationale for the selection of targets.

Moreover, because rTMS and modified response-prevention protocol were introduced as a combined treatment package, the contribution of the individual treatment components cannot be separated. Accordingly, the contribution of this report is the operational description and initial feasibility assessment of an integrated clinical protocol, rather than evidence of efficacy or superiority. In addition, the combined rTMS-modified response-prevention sessions were administered on weekdays, and continuity of exposure-related practice outside these structured sessions was not systematically documented. Similarly, patients’ subjective experience of treatment feasibility and their willingness to recommend this approach to other patients were not systematically assessed using structured instruments. Both patients presented with severe OCD symptoms that had shown insufficient response to prior treatment, which likely contributed to their high motivation to pursue an intensive protocol. Future studies should incorporate structured assessments of motivation and patient-reported acceptability to better characterize the feasibility of this intensive multimodal treatment approach in clinical practice. Within these limitations, the report provides a reproducible protocol specification that can inform prospective controlled studies.

Future controlled trials with sufficient sample sizes and harmonized outcome assessment are needed to evaluate this multimodal approach systematically. Such trials could clarify whether a structured combination of multi-target rTMS and ERP offers added value for patients with severe or treatment-resistant OCD and could help determine which patient subgroups are most likely to benefit. Beyond larger-scale replication, dismantling designs would further help clarify the relative contribution of individual protocol components: for example, comparing the three-target sequential protocol used here against a reduced two-target approach (e.g., left DLPFC and SMA only), evaluating whether a shorter course of 20 sessions yields comparable benefit to the 30 sessions applied in this report, contrasting rTMS alone against the modified response-prevention component alone, and examining whether the timing of exposure relative to stimulation influences treatment response. More frequent longitudinal assessments, such as weekly Y-BOCS and BDI-II ratings rather than pre- and post-treatment measurement alone, would additionally allow finer-grained characterization of the symptom trajectory.

In summary, the contribution of this report lies in its explicit protocol architecture: a repeated two-session-per-day, three-target TBS schedule integrated with a clinician-supervised, largely self-administered exposure procedure using cue-terminated response prevention. Its feasibility and tolerability in two patients with severe OCD, together with the observed clinical course, support prospective controlled evaluation; no conclusions regarding efficacy or component-specific effects can be drawn.

Patient perspective (Case 1):

The patient reported significantly less time spent on compulsive rituals and improved cognitive control over these rituals. She described the burden of her remaining obsessive–compulsive symptoms as considerably reduced and stated, verbatim, that the remaining symptoms were “barely relevant to everyday life” and that this enabled her to organize her daily activities noticeably better.

Patient perspective (Case 2):

The patient also reported a markedly reduced amount of time spent on compulsive rituals and described feeling generally calmer and less burdened. He required considerably less reassurance from others in daily life, and he too described the control rituals as less automatic and more manageable. In addition, he reported being able to think about future perspectives again.

## Figures and Tables

**Table 1:** Clinical Timeline—Patient 1.

Date/Time Point	Event
2022	Onset of OCD symptoms
Since 12/2024	Multiple psychiatric inpatient admissions
Until 07/2025	Conventional ERP (outpatient/inpatient); exact start date not documented; discontinued without clinically meaningful improvement.
12/2024–03/2025	Paroxetine (≤60 mg/day); discontinued due to insufficient benefit and at the patient’s request.
Since 03/2025	Clomipramine (225 mg/day); ongoing, continued during index protocol.
Since 06/2025	Risperidone (2 mg/day); ongoing, continued during index protocol.
Baseline	Y-BOCS/BDI-II assessment
11/2025	Initiation of rTMS with modified response-prevention protocol.
20th treatment unit	Y-BOCS interim assessment
End of treatment	Y-BOCS/BDI-II assessment
+4 weeks	Y-BOCS follow-up assessment

Note 1. Dates are reconstructed retrospectively from medical records. Note 2. Medication doses remained largely stable during the index treatment period; the risperidone dose was reduced from 2 mg/day to 1.5 mg/day during the final sessions. There were no other medication changes during treatment or during the subsequent four-week follow-up period. Note 3. No information on pharmacological treatment prior to 2024 was available in the records reviewed; the interval between symptom onset (2022) and the first documented pharmacological intervention reflects a gap in the available records and does not represent a confirmed untreated phase.

**Table 2:** Clinical Timeline—Patient 2.

Date/Time Point	Event
2020	Onset of depression with obsessive thoughts (without compulsive behaviors).
Since 2020	Multiple psychiatric inpatient admissions across several clinics.
2020	Escitalopram (15 mg/day); discontinued due to side effects.
2020	Risperidone (1.5 mg/day) as augmentation; discontinued due to side effects.
2024	Mirtazapine; discontinued due to side effects
Before 05/2025	Amisulpride; discontinued due to pronounced hyperprolactinemia.
Before 05/2025	Sertraline, discontinued due to side effects and insufficient efficacy; trazodone (200 mg/day), subsequently discontinued.
03/2025–07/2025	Quetiapine (up to 250 mg/day) as augmentation and for management of inner tension and sleep disturbances; subsequently discontinued.
06/2025	Emergence of pronounced checking compulsions with marked functional impairment.
Since 05/2025	Lithium (1350 mg/day, i.e., 675 mg twice daily; serum level 0.5–0.7 mmol/L); ongoing, continued during index protocol.
Since 05/2025	Amitriptyline (200 mg/day); ongoing, continued during index protocol
(During current inpatient admission) No date documented.	Conventional CBT with exposure was documented during the current inpatient admission, whereas the exact start date is not documented. At the time rTMS with the modified response-prevention protocol was initiated, no structured ERP was ongoing.
Since 08/01/2026	Aripiprazole (15 mg/day) as augmentation; ongoing, continued during index protocol.
Baseline	Y-BOCS/BDI-II assessment
28/01/2026	Initiation of rTMS with modified response-prevention protocol.
20th treatment unit	Y-BOCS assessment
End of treatment	Y-BOCS/BDI-II assessment
+4 weeks	Y-BOCS follow-up assessment

Note 1. Dates are reconstructed retrospectively from medical records. Note 2. Medication doses remained largely stable during the index treatment period; the amitriptyline dose was increased from 150 mg/day to 200 mg/day over the course of treatment. There were no other medication changes during treatment or during the subsequent four-week follow-up period.

**Table 3:** Clinical outcomes during multimodal treatment (rTMS plus the modified response-prevention protocol).

Patient	Scale	Baseline	After 20 Treatment Units	After 30 Treatment Units	4-Week Follow-Up	Reduction from Baseline to Post-Treatment (%)
**1**	**BDI-II**	50	15	8	NA	84.0
	**Y-BOCS**	29	12	11	9	62.1
**2**	**BDI-II**	37	NA	20	NA	45.9
	**Y-BOCS**	29	11	10	13	65.5

Note. Reduction from baseline to post-treatment (%) = percentage reduction from baseline to end of treatment (after 30 treatment units). One treatment unit consisted of two rTMS sessions separated by a 60-min inter-session interval. NA = not assessed. BDI-II = Beck Depression Inventory-II; Y-BOCS = Yale–Brown Obsessive–Compulsive Scale.

## Data Availability

The underlying clinical data for this case report are not publicly available and cannot be shared, as they contain sensitive, potentially identifiable patient-level information. All data relevant to the interpretation of the cases are included in the article.

## Abbreviations

The following abbreviations are used in this manuscript:

Abbreviation	Full term
BDI-II	Beck Depression Inventory-II
CBT	Cognitive Behavioral Therapy
CSTC	Cortico-Striato-Thalamo-Cortical
cTBS	Continuous Theta-Burst Stimulation
DLPFC	Dorsolateral Prefrontal Cortex
DMN	Default Mode Network
EEG	Electroencephalography
ERP	Exposure and Response Prevention
FPN	Frontoparietal Network
HF-rTMS	High-Frequency Repetitive Transcranial Magnetic Stimulation
IFG	Inferior Frontal Gyrus
iTBS	Intermittent Theta-Burst Stimulation
L-DLPFC	Left Dorsolateral Prefrontal Cortex
LF-rTMS	Low-Frequency Repetitive Transcranial Magnetic Stimulation
LTD	Long-Term Depression
LTP	Long-Term Potentiation
MRI	Magnetic Resonance Imaging
NA	Not Assessed
OCD	Obsessive–Compulsive Disorder
pre-SMA	Pre-Supplementary Motor Area
R-DLPFC	Right Dorsolateral Prefrontal Cortex
RCT	Randomized Controlled Trial
RMT	Resting Motor Threshold
rTMS	Repetitive Transcranial Magnetic Stimulation
SMA	Supplementary Motor Area
SN	Salience Network
SSRI	Selective Serotonin Reuptake Inhibitor
SSRT	Stop-Signal Reaction Time
TBS	Theta-Burst Stimulation
TMS	Transcranial Magnetic Stimulation
Y-BOCS	Yale–Brown Obsessive–Compulsive Scale

## References

[1] Stein D.J., Costa D.L.C., Lochner C., Miguel E.C., Reddy Y.C.J., Shavitt R.G., van den Heuvel O.A., Simpson H.B. (2019). Obsessive–Compulsive Disorder. Nat. Rev. Dis. Primers.

[2] Brock H., Rizvi A., Hany M. (2024). Obsessive-Compulsive Disorder. StatPearls [Internet].

[3] Cheng S., Chen Z., Wu C., Li X., Shen X., Qiu R., Tang N., Feng C., Wang W., Lv J. (2025). The Comorbidity of Anxiety and Depression Symptoms in Obsessive–Compulsive Disorder: A Network Analysis. Front. Psychiatry.

[4] Overbeek T., Schruers K., Vermetten E., Griez E. (2002). Comorbidity of Obsessive-Compulsive Disorder and Depression: Prevalence, Symptom Severity, and Treatment Effect. J. Clin. Psychiatry.

[5] Reid J.E., Laws K.R., Drummond L., Vismara M., Grancini B., Mpavaenda D., Fineberg N.A. (2021). Cognitive Behavioural Therapy with Exposure and Response Prevention in the Treatment of Obsessive-Compulsive Disorder: A Systematic Review and Meta-Analysis of Randomised Controlled Trials. Compr. Psychiatry.

[6] Cohen S.E., Storosum B.W., Zantvoord J.B., Mattila T.K., de Boer A., Denys D. (2025). Individual Patient Data Meta-Analysis of Placebo-Controlled Trials of Selective Serotonin Reuptake Inhibitors Submitted for Regulatory Approval in Adult Obsessive–Compulsive Disorder. Br. J. Psychiatry.

[7] Swierkosz-Lenart K., Dos Santos J.F.A., Elowe J., Clair A.-H., Bally J.F., Riquier F., Bloch J., Draganski B., Clerc M.-T., Pozuelo Moyano B. (2023). Therapies for Obsessive-Compulsive Disorder: Current State of the Art and Perspectives for Approaching Treatment-Resistant Patients. Front. Psychiatry.

[8] Mao L., Hu M., Luo L., Wu Y., Lu Z., Zou J. (2022). The Effectiveness of Exposure and Response Prevention Combined with Pharmacotherapy for Obsessive-Compulsive Disorder: A Systematic Review and Meta-Analysis. Front. Psychiatry.

[9] Pittenger C., Bloch M.H. (2014). Pharmacological Treatment of Obsessive-Compulsive Disorder. Psychiatr. Clin. N. Am..

[10] Del Casale A., Sorice S., Padovano A., Simmaco M., Ferracuti S., Lamis D.A., Rapinesi C., Sani G., Girardi P., Kotzalidis G.D. (2019). Psychopharmacological Treatment of Obsessive-Compulsive Disorder (OCD). Curr. Neuropharmacol..

[11] Dell’Osso B. (2023). Defining and Understanding Treatment Resistance in Obsessive-Compulsive Disorder. Eur. Psychiatry.

[12] Albert U., Salvo G.D., Solia F., Rosso G., Maina G. (2018). Combining Drug and Psychological Treatments for Obsessive- Compulsive Disorder: What is the Evidence, When and for Whom. Curr. Med. Chem..

[13] Fitzgerald P.B., George M.S., Pridmore S. (2022). The Evidence Is in: Repetitive Transcranial Magnetic Stimulation Is an Effective, Safe and Well-Tolerated Treatment for Patients with Major Depressive Disorder. Aust. N. Z. J. Psychiatry.

[14] Chen L., Klooster D.C.W., Tik M., Thomas E.H.X., Downar J., Fitzgerald P.B., Williams N.R., Baeken C. (2023). Accelerated Repetitive Transcranial Magnetic Stimulation to Treat Major Depression: The Past, Present and Future. Harv. Rev. Psychiatry.

[15] Grassi G., Moradei C., Cecchelli C. (2023). Will Transcranial Magnetic Stimulation Improve the Treatment of Obsessive–Compulsive Disorder? A Systematic Review and Meta-Analysis of Current Targets and Clinical Evidence. Life.

[16] Tadayonnejad R., Wilson A.C., Corlier J., Lee J.C., Ginder N.D., Levitt J.G., Wilke S.A., Marder K.G., Krantz D., Bari A.A. (2020). Sequential Multi-Locus Transcranial Magnetic Stimulation for Treatment of Obsessive-Compulsive Dis-order with Comorbid Major Depression: A Case Series. Brain Stimul..

[17] Tadayonnejad R., Wilson A.C., Chu S.A., Corlier J., Citrenbaum C., Ngo T.D.P., Hovhannisyan E., Ginder N.D., Levitt J.G., Wilke S.A. (2022). Use of Right Orbitofrontal Repetitive Transcranial Magnetic Stimulation (rTMS) Aug-mentation for Treatment-Refractory Obsessive-Compulsive Disorder with Comorbid Major Depressive Disorder. Psychiatry Res..

[18] Posner J., Marsh R., Maia T.V., Peterson B.S., Gruber A., Simpson H.B. (2013). Reduced Functional Connectivity within the Limbic Cortico-striato-thalamo-cortical Loop in Unmedicated Adults with Obsessive-compulsive Disorder. Hum. Brain Mapp..

[19] Calzà J., Gürsel D.A., Schmitz-Koep B., Bremer B., Reinholz L., Berberich G., Koch K. (2019). Altered Cortico–Striatal Functional Connectivity During Resting State in Obsessive–Compulsive Disorder. Front. Psychiatry.

[20] Bijanki K.R., Pathak Y.J., Najera R.A., Storch E.A., Goodman W.K., Simpson H.B., Sheth S.A. (2021). Defining Functional Brain Networks Underlying Obsessive–Compulsive Disorder (OCD) Using Treatment-Induced Neuroimaging Changes: A Systematic Review of the Literature. J. Neurol. Neurosurg. Psychiatry.

[21] Rehn S., Eslick G.D., Brakoulias V. (2018). A Meta-Analysis of the Effectiveness of Different Cortical Targets Used in Repetitive Transcranial Magnetic Stimulation (rTMS) for the Treatment of Obsessive-Compulsive Disorder (OCD). Psychiatr. Q..

[22] Ji G., Xie W., Yang T., Wu Q., Sui P., Bai T., Chen L., Chen L., Chen X., Dong Y. (2021). Pre-supplementary Motor Network Connectivity and Clinical Out-come of Magnetic Stimulation in Obsessive–Compulsive Disorder. Hum. Brain Mapp..

[23] Liang K., Li H., Bu X., Li X., Cao L., Liu J., Gao Y., Li B., Qiu C., Bao W. (2021). Efficacy and Tolerability of Repetitive Transcranial Magnetic Stimulation for the Treatment of Obsessive-Compulsive Disorder in Adults: A Systematic Re-view and Network Meta-Analysis. Transl. Psychiatry.

[24] Khedr E.M., Elbeh K., Saber M., Abdelrady Z., Abdelwarith A. (2022). A Double Blind Randomized Clinical Trial of the Effectiveness of Low Frequency rTMS over Right DLPFC or OFC for Treatment of Obsessive-Compulsive Disorder. J. Psychiatr. Res..

[25] Launes G., Hagen K., Sunde T., Öst L.-G., Klovning I., Laukvik I.-L., Himle J.A., Solem S., Hystad S.W., Hansen B. (2019). A Randomized Controlled Trial of Concentrated ERP, Self-Help and Waiting List for Obsessive- Compulsive Disor-der: The Bergen 4-Day Treatment. Front. Psychol..

[26] Skjold S.H., Hagen K., Wheaton M.G., Hjelle K.M., Björgvinsson T., Hansen B. (2024). The Effectiveness and Acceptability of the Bergen 4-Day Treatment for Adolescents with OCD: A Replication and Extension. BMC Psychiatry.

[27] Law C., Boisseau C.L. (2019). Exposure and Response Prevention in the Treatment of Obsessive-Compulsive Disorder: Current Perspectives. Psychol. Res. Behav. Manag..

[28] Yan J., Cui L., Wang M., Cui Y., Li Y., Yan J., Cui L., Wang M., Cui Y., Li Y. (2022). The Efficacy and Neural Correlates of ERP-Based Therapy for OCD & TS: A Systematic Review and Meta-Analysis. J. Integr. Neurosci..

[29] Huang Y., Yang H., Zhu C., Jiang X., Zhu W., Liang Y., Ma L., Wang Y., Tang W. (2022). An Exploratory Study of a Novel Combined Therapeutic Modality for Obses-sive-Compulsive Disorder. Brain Sci..

[30] Chou P.-H., Sack A.T., Su K.-P. (2022). Targeting Three Brain Regions (Bilateral SMA, Left and Right DLPFC) Sequentially in One Session Using Combined Repetitive Transcranial Magnetic Stimulation and Intermittent Theta-Burst Stimulation in Treatment-Refractory Obsessive-Compulsive Disorder: A Case Report. Clin. Psychopharmacol. Neurosci..

[31] Pallanti S., Hollander E., Bienstock C., Koran L., Leckman J., Marazziti D., Pato M., Stein D., Zohar J. (2002). International Treatment Refractory OCD Consortium. Treatment Non-Response in OCD: Methodological Issues and Operational Definitions. Int. J. Neuropsychopharmacol..

[32] Provaznikova B., Monn A., Seifritz E., Kronenberg G., Olbrich S. (2025). EEG Alpha Activity as Predictor for TBS-rTMS Treatment Outcome in Depression. J. Psychiatr. Res..

[33] Trapp N.T., Purgianto A., Taylor J.J., Singh M.K., Oberman L.M., Mickey B.J., Youssef N.A., Solzbacher D., Zebley B., Cabrera L.Y. (2025). Consensus Review and Considerations on TMS to Treat Depression: A Comprehensive Update Endorsed by the National Network of Depression Centers, the Clinical TMS Socie-ty, and the International Federation of Clinical Neurophysiology. Clin. Neuro-Physiol..

[34] Turi Z., Lenz M., Paulus W., Mittner M., Vlachos A. (2021). Selecting Stimulation Intensity in Repetitive Transcranial Magnetic Stimulation Studies: A Systematic Review between 1991 and 2020. Eur. J. Neurosci..

[35] Rossi S., Hallett M., Rossini P.M., Pascual-Leone A. (2009). Safety, Ethical Considerations, and Application Guidelines for the Use of Transcranial Magnetic Stimulation in Clinical Practice and Research. Clin. Neurophysiol..

[36] Rossi S., Antal A., Bestmann S., Bikson M., Brewer C., Brockmöller J., Car-penter L.L., Cincotta M., Chen R., Daskalakis J.D. (2021). Safety and Recommendations for TMS Use in Healthy Subjects and Patient Populations, with Up-dates on Training, Ethical and Regulatory Issues: Expert Guidelines. Clin. Neurophysiol..

[37] Diao X., Lu Q., Qiao L., Gong Y., Lu X., Feng M., Su P., Shen Y., Yuan T.-F., He C. (2022). Cortical Inhibition State-Dependent iTBS Induced Neural Plasticity. Front. Neurosci..

[38] Wang L., Zhou Q.-H., Wang K., Wang H.-C., Hu S.-M., Yang Y.-X., Lin Y.-C., Wang Y.-P. (2022). Frontoparietal Paired Associative Stimulation versus Single-Site Stimulation for Generalized Anxiety Disorder: A Pilot rTMS Study. J. Psychiatry Neurosci..

[39] Cerins A., Thomas E.H.X., Barbour T., Taylor J.J., Siddiqi S.H., Trapp N., McGirr A., Caulfield K.A., Brown J.C., Chen L. (2024). A New Angle on Transcranial Magnetic Stimulation Coil Orientation: A Targeted Narrative Review. Biol. Psychiatry Cogn. Neurosci. Neuroimaging.

[40] Gomes P.V.O., Brasil-Neto J.P., Allam N., Rodrigues de Souza E. (2012). A Randomized, Double-Blind Trial of Repetitive Transcranial Magnetic Stimulation in Obsessive-Compulsive Disorder With Three-Month Follow-Up. J. Neuropsychiatry Clin. Neurosci..

[41] Veale D., Roberts A. (2014). Obsessive-Compulsive Disorder. BMJ.

[42] Saxena S., Brody A.L., Schwartz J.M., Baxter L.R. (1998). Neuroimaging and Frontal-Subcortical Circuitry in Obsessive-Compulsive Disorder. Br. J. Psychiatry Suppl..

[43] Hawken E.R., Dilkov D., Kaludiev E., Simek S., Zhang F., Milev R. (2016). Transcranial Magnetic Stimulation of the Supplementary Motor Area in the Treatment of Obsessive-Compulsive Disorder: A Multi-Site Study. Int. J. Mol. Sci..

[44] Moreira P.S., Marques P., Soriano-Mas C., Magalhães R., Sousa N., Soares J.M., Morgado P. (2017). The Neural Correlates of Obsessive-Compulsive Disorder: A Multimodal Perspective. Transl. Psychiatry.

[45] Xu Y., Zhang Y. (2023). Abnormal Voxel-Mirrored Homotopic Connectivity in First-Episode, Drug-Naïve Patients with Obsessive–Compulsive Disorder. Eur. J. Neurosci..

[46] Bruin W.B., Abe Y., Alonso P., Anticevic A., Backhausen L.L., Balachander S., Bargallo N., Batistuzzo M.C., Benedetti F., Bertolin Triquell S. (2023). The Functional Connectome in Obsessive-Compulsive Disorder: Resting-State Mega-Analysis and Machine Learning Classification for the ENIGMA-OCD Consortium. Mol. Psychiatry.

[47] Perera M.P.N., Gotsis E.S., Bailey N.W., Fitzgibbon B.M., Fitzgerald P.B. (2024). Exploring Functional Connectivity in Large-Scale Brain Networks in Obsessive-Compulsive Disorder: A Systematic Review of EEG and fMRI Studies. Cerebral. Cortex..

[48] Gao J., Zhou Y., Yang X., Luo J., Meng F., Zheng D., Li Z. (2019). Abnormalities with-in and beyond the Cortico-Striato-Thalamo-Cortical Circuitry in Medication-Free Patients with OCD Revealed by the Fractional Amplitude of Low-Frequency Fluctuations and Resting-State Functional Connectivity. Neurosci. Lett..

[49] Zhu Y., Jiang X., Ji W. (2018). The Mechanism of Cortico-Striato-Thalamo-Cortical Neurocircuitry in Response Inhibition and Emotional Responding in Attention Deficit Hyperactivity Disorder with Comorbid Disruptive Behavior Disorder. Neurosci. Bull..

[50] Ahmari S.E., Rauch S.L. (2022). The Prefrontal Cortex and OCD. Neuropsychopharmacology.

[51] Schutter D.J.L.G. (2009). Antidepressant Efficacy of High-Frequency Transcranial Magnetic Stimulation over the Left Dorsolateral Prefrontal Cortex in Double-Blind Sham-Controlled Designs: A Meta-Analysis. Psychol. Med..

[52] Vaghi M.M., Vértes P.E., Kitzbichler M.G., Apergis-Schoute A.M., Van Der Flier F.E., Fineberg N.A., Sule A., Zaman R., Voon V., Kundu P. (2017). Specific Frontostriatal Circuits for Impaired Cognitive Flexibility and Goal-Directed Planning in Obsessive-Compulsive Disorder: Evidence From Resting-State Functional Connectivity. Biol. Psychiatry.

[53] Plewnia C., Pasqualetti P., Große S., Schlipf S., Wasserka B., Zwissler B., Fallgatter A. (2014). Treatment of Major Depression with Bilateral Theta Burst Stimulation: A Randomized Controlled Pilot Trial. J. Affect. Disord..

[54] Plewnia C., Brendel B., Schwippel T., Nieratschker V., Ethofer T., Kammer T., Padberg F., Martus P., Fallgatter A.J. (2021). Treatment of Major Depressive Disorder with Bilateral Theta Burst Stimulation: Study Protocol for a Randomized, Double-Blind, Placebo-Controlled Multicenter Trial (TBS-D). Eur. Arch. Psychiatry Clin. Neurosci..

[55] Hazari N., Narayanaswamy J.C., Venkatasubramanian G. (2019). Neuroimaging Findings in Obsessive–Compulsive Disorder: A Narrative Review to Elucidate Neurobiological Underpinnings. Indian J. Psychiatry.

[56] Elbeh K.A.M., Elserogy Y.M.B., Khalifa H.E., Ahmed M.A., Hafez M.H., Khedr E.M. (2016). Repetitive Transcranial Magnetic Stimulation in the Treatment of Obsessive-Compulsive Disorders: Double Blind Randomized Clinical Trial. Psychiatry Res..

[57] Nachev P., Wydell H., O’Neill K., Husain M., Kennard C. (2007). The Role of the Pre-Supplementary Motor Area in the Control of Action. Neuroimage.

[58] Tomiyama H., Murayama K., Nemoto K., Tomita M., Hasuzawa S., Mizobe T., Kato K., Ohno A., Tsuruta S., Togao O. (2021). Increased Functional Connectivity between Presupplementary Motor Area and Inferior Frontal Gyrus Associated with the Ability of Motor Response Inhibition in Obsessive–Compulsive Disorder. Hum. Brain Mapp..

[59] Chambers C.D., Garavan H., Bellgrove M.A. (2009). Insights into the Neural Basis of Response Inhibition from Cognitive and Clinical Neuroscience. Neurosci. Biobehav. Rev..

[60] Singh K.R., Bhattacharya D., Sharma R.M., Chauhan V.S., Agrawal M., Yadav P., Panda S.P., Sharma N. (2025). Efficacy of Repetitive Transcranial Magnetic Stimulation as an Adjunctive Treatment in Resistant Obsessive–Compulsive Disorder: A Randomized Controlled Trial. Ind. Psychiatry J..

[61] Alizadehgoradel J., Molaei B., Barzegar Jalali K., Pouresmali A., Sharifi K., Hallajian A.-H., Nejati V., Glinski B., Vicario C.M., Nitsche M.A. (2024). Targeting the Prefrontal-Supplementary Motor Network in Obsessive-Compulsive Disorder with Intensified Electrical Stimulation in Two Dosages: A Randomized, Con-trolled Trial. Transl. Psychiatry.

[62] Dehghani-Arani F., Kazemi R., Hallajian A.-H., Sima S., Boutimaz S., He-dayati S., Koushamoghadam S., Safarifard R., Salehinejad M.A. (2024). Metaanalysis of Repetitive Transcranial Magnetic Stimulation (rTMS) Efficacy for OCD Treatment: The Impact of Stimulation Parameters, Symptom Subtype and rTMS-Induced Electrical Field. J. Clin. Med..

[63] Ribeiro Coutinho N.L.C., Lopes G.V.F., Caparelli-Dáquer E., Scotti-Muzzi E. (2025). Multi-Target Theta Burst Stimulation (cTBS) as an Adjunctive Strategy for Refractory Obsessive-Compulsive and Major Depressive Disorders: A Case Report. Transcranial. Magn. Stimul..

[64] van Rooij S.J.H., Arulpragasam A.R., McDonald W.M., Philip N.S. (2024). Accelerated TMS—Moving Quickly into the Future of Depression Treatment. Neuropsychopharmacology.

[65] Fitzsimmons S.M.D.D., Postma T.S., van Campen A.D., Vriend C., Batelaan N.M., van Oppen P., Hoogendoorn A.W., van der Werf Y.D., van den Heuvel O.A. (2025). Transcranial Magnetic Stimulation–Induced Plasticity Improving Cognitive Control in Obsessive-Compulsive Disorder, Part I: Clinical and Neuroimaging Outcomes From a Randomized Trial. Biol. Psychiatry.

[66] Hamada M., Hanajima R., Terao Y., Okabe S., Nakatani-Enomoto S., Fu-rubayashi T., Matsumoto H., Shirota Y., Ohminami S., Ugawa Y. (2009). Primary Motor Cortical Metaplasticity Induced by Priming over the Supplementary Motor Area. J. Physiol..

[67] Deng J., Fang W., Gong Y., Bao Y., Li H., Su S., Sun J., Shi J., Lu L., Shi L. (2021). Augmentation of Fear Extinction by Theta-Burst Transcranial Magnetic Stimulation of the Prefrontal Cortex in Humans. J. Psychiatry Neurosci..

[68] (1997). Obsessive-Compulsive Cognitions Working Group. Cognitive Assessment of Obsessive-Compulsive Disorder. Behav. Res. Ther..

[69] Taren A.A., Gianaros P.J., Greco C.M., Lindsay E.K., Fairgrieve A., Brown K.W., Rosen R.K., Ferris J.L., Julson E., Marsland A.L. (2015). Mindfulness Meditation Training Alters Stress-Related Amygdala Resting State Functional Connectivity: A Randomized Controlled Trial. Soc. Cogn. Affect Neurosci..

[70] Mathur S., Sharma M.P., Balachander S., Kandavel T., Reddy Y.J. (2021). A Randomized Controlled Trial of Mindfulness-Based Cognitive Therapy vs Stress Management Training for Obsessive-Compulsive Disorder. J. Affect. Disord..

[71] de Wit S.J. (2012). Presupplementary Motor Area Hyperactivity During Response Inhibition: A Candidate Endophenotype of Obsessive-Compulsive Disorder. Am. J. Psychiatry.

[72] McLaughlin N.C.R., Kirschner J., Foster H., O’Connell C., Rasmussen S.A., Greenberg B.D. (2016). Stop Signal Reaction Time Deficits in a Lifetime Obsessive-Compulsive Disorder Sample. J. Int. Neuropsychol. Soc..

[73] Ravid A., Collins L., Coles M.E. (2020). “Not Just Right Experiences” in Children and Adolescents: Phenomenology and Relation to OCD Symptoms. J. Obs.-Compuls. Relat. Disord..

[74] Horncastle T., Ludlow A.K., Gutierrez R. (2022). Not Just Right Experiences and In-completeness as a Predictor of Obsessive Compulsive Symptoms in Clinical and Community Samples: A Meta-Analysis. J. Obs.-Compuls. Relat. Disord..

[75] Szechtman H., Woody E. (2004). Obsessive-Compulsive Disorder as a Disturbance of Security Motivation. Psychol. Rev..

[76] Zor R., Szechtman H., Hermesh H., Fineberg N.A., Eilam D. (2011). Manifestation of Incompleteness in Obsessive-Compulsive Disorder (OCD) as Reduced Functionality and Extended Activity beyond Task Completion. PLoS ONE.

[77] Ensuring Consent for Publishing Medical Case Reports. https://publicationethics.org/guidance/guideline/ensuring-consent-publishing-medical-case-reports.

